# Surgical Report on Those Affections of the Penis Which Are Generally Considered as Primary Symptoms of Syphilis

**Published:** 1819-07-01

**Authors:** 


*Surgical Report on those Affections of the Penis which are generally considered as primary Symptoms of Syphilis.* By C. H. Todd, Surgeon to the Richmond Surgical Hospital.

(Dublin Hospital Reports, vol. ii.)

In the Richmond Surgical Hospital seven small wards are set apart for the reception of male patients affected with venereal complaints. Mr. S. has had a large proportion of these cases under his care, and proposes, in a series of papers, to report on the various appearances, progress, and treatment of these affections, divested of all theoretical discussions, and strictly confined to a statement of facts. Nothing can be more proper or judicious than such a proceeding, and we shall now lay before the English reader a condensed outline of Mr. Todd's first Report.

§ I. *Inflammation of the Penis.* This, whether arising from neglected secretions, improperly treated excoriations, gonorrhœa, ulcers, or derangement of the general health, is very common, especially under the form of phymosis or paraphymosis. Acute inflammation of the penis is very readily excited, and very rapidly produces mortification, even so early as the fourth or fifth day. This reflection ought to lead to the most active treatment. Confinement to bed; rigid abstinence; free depletion; especially copious venesection, repeated twice or thrice. The local abstraction by leeches must be looked upon as purely auxiliary. Cold and warm fomentations are equally beneficial, if equally grateful to the patient. The cold may be directed *first*, and continued as long as it affords relief; when it ceases to do so, or excites uneasiness, warm stupes or poultices are to be substituted. Tepid injections are to be thrown in between the gland and prepuce; and if the phymosis be severe, with extensive excoriations and considerable discharge, the foreskin is to be slit up about an inch.

The inspection of the ulcers underneath does not always remove doubt as to their real nature. Mr. Todd thinks that, in some constitutions, genuine chancres will heal spontaneously; or their specific characters will be altered by *severe inflammation,*—in some instances effaced. In the Richmond hospital, the surgeons have long been in the habit of abstaining from the exhibition of mercury in cases where, on the subsidence of the inflammation, no ulcer was discovered, or where existing ulcers presented a florid, healthy appearance, and speedily cicatrized under simple treatment. Where these ulcerations, however, resist local treatment, of different kinds, and particularly if any symptom of bubo should occur, the exhibition of mercury is commenced, "which seldom fails in producing salutary effects on the disease, provided its effects on the constitution be not obstructed by any peculiarity of habit, or irregularity in the employment of the remedy." 155.

The morbid state of the prepuce, he considers, is often connected with derangement of the general health, but particularly of the digestive organs; in which cases, calomel, in pretty large doses, is given with great advantage. If it be objected to this practice, that the constitution becomes quickly mercurialized, and the question of syphilitic or pseudo-syphilitic origin is thereby left in doubt, Mr. T. answers, that although these cases of chronic inflammation of the penis are rarely syphilitic, yet whether they are or are not so, the *practice* is the best that can be employed. This plan has never, in the hospital, or in private practice, been followed by constitutional, or other unpleasant symptoms.

We must pass over paraphymosis and erysipelas of the penis for the sake of dwelling a little on gangrene of that organ, than which occurrence, there are few which excite more alarm in the mind of the patient, or anxiety in that of the practitioner.

When a patient applies at the hospital with the extremity of the prepuce mortified, and the rest of the penis highly inflamed, the treatment is strictly antiphlogistic; the diseased parts are fomented at regular intervals, and the effervescing poultice applied every third or fourth hour. When the patient complains of the weight of the poultice, dossils of lint wet in the nitrous or muriatic acid lotion are found very efficacious in correcting the fœtor, and promoting the separation of the sloughs. When sloughs are separating from the corpora cavernosa and glans penis, the case is often rendered very embarrassing by hæmorrhage, which sometimes threatens the life of the patient. Here we must endeavour to ascertain whether the hæmorrhage be from a single vessel, or from a diseased surface. If phymosis exist, as is commonly the case, the prepuce must be laid open and retracted, so as to expose the ulcer. We are sometimes fortunate enough to discover the orifice of the artery, and may secure it with the forceps and ligature; but more generally the blood seems to issue from a number of points, or the bleeding vessel is so deeply situated within the surrounding granulations that it cannot be distinguished. Here dossils of lint dipped in ol. terebinth. may be fixed on the part by means of a bandage or sponge. To apply pressure with advantage, an elastic catheter is to be introduced into the bladder; and having regulated the application and compresses on the diseased surface, we must apply a long and narrow roller to the penis, with that degree of tightness which may moderate the flux of blood into the part, without producing pain. If this be kept wet with very cold water and a little spirit, it will be more effectual. The parts are to be allowed to remain in this state for two, three, or four days, according to circumstances. If all means fail, we must cut upon the dorsal arteries of the penis near to the pubes, where they lie parallel to each other, and inclose them in a ligature.

### General Remarks.

Every one in extensive hospital or private practice must have observed the almost infinite variety of forms which ulcers on the penis assume, and how small the proportion of well defined, or as they have been termed, Hunterian chancres, when compared with ulcers of a different description; in short, ulcers of a very irregular form, exhibiting none of the characters of chancre, are so common, can be so satisfactorily traced to venereal origin, and are so generally succeeded by constitutional symptoms of syphilis, that all attempts to systematize or arrange their appearances, consistent with practical observation, will be found entirely futile in the present state of our knowledge.

The external male organs of generation consist of so many different kinds of structure, that it is reasonable to expect a corresponding variety in the character of the ulcers affecting them. Thus a chancre on the glans differs from the same affection on the prepuce; and a chancre on the frenum or internal surface of the prepuce differs very considerably from a chancre on its external surface, or on the scrotum. These facts were strongly stated by Mr. Hunter, but his observations have not been sufficiently remembered by modern practitioners. Again, if the venereal poison be applied to a sound part, the chancre may assume what is considered as its legitimate form, and its progress will be slow; but if the virus come in contact with a raw surface—if, in the act of coition, the frenum or edge of the prepuce be lacerated—if there be an excoriation, an herpetic eruption, or any other cutaneous disease or ulcer of the penis, the application of poisonous matter to such surfaces may be expected to give rise to appearances, in which none of the nominal characters of chancre may be recognized, and to which inflammation and phagedenic ulceration will be very likely to succeed. The age and habit of the patient, also greatly vary the external appearance of chancres; and so will the stage of the disease in the female who communicates it.

As it is Mr. Todd's intention, in the next volume, to take up the treatment of venereal sores in the Richmond Hospital, he does not enter much on it here, but we may lay the following passage from a man of great experience before our Readers, as worthy their notice.

"Recent publications, however, induce me to enter my protest against the conclusion that we ought to discard mercury as an anti-venereal medicine; these publications seem to me only to prove, that mercury has been too often injudiciously administered. We often hear of the spontaneous cure of syphilis, and we occasionally meet with instances in which venereal diseases will go through a certain course, and then disappear, without the aid of mercury; but such efforts of the constitution, or modifications of these complaints, which are irregular, and hitherto not fully explained, ought not to influence our general practice." 185.

There is no doubt but that mercury is capable of exciting a deleterious as well as salutary influence on the system, according as it is judiciously or injudiciously managed. When the process is salutary, a peculiar fever is excited, accompanied with thirst, head-ache, and frequency of pulse; the salivary glands become enlarged, and their secretion considerably increased; during all which, the venereal symptoms gradually disappear. But, on the other hand, when the constitution of the patient resists this action, which is very often the case, if mercury be persevered in, it will have no effect on the disease, or it will impart to it new properties and characters, which may render it more obstinate and untractable.

The state of the system *here* is very different from that described above. The patient becomes languid and oppressed, with sighing, palpitation, and startings from sleep; he has no desire for food; his pulse becomes frequent, and sometimes irregular. The salivary glands are but little, if at all, excited; a slight degree of soreness takes place in the gums, or they become spongy, and ulcerated along their margins; and it is very remarkable that this affection of the gums will often entirely subside, return, and again subside, at irregular intervals, although mercury has been continued without interruption, or perhaps administered in augmented doses, and in the most active forms.

When the deleterious effect of mercury has been once established in the system, it is surprizing how long it will continue to operate. Mr. Todd has detected it in patients who had not taken a single grain of that medicine for several years; the mercurial action will even cease entirely for a time, and be called forth by exposure to cold, by privations, or by intemperance; and persons thus affected will experience the same sensations as if they were actually using mercury, and on the eve of salivation.

These things should be strictly attended to by the practitioner; he should administer mercury with a frugal hand, when he finds it disagree, and persevere steadily when he finds it useful.

"By adhering to this rule, for some years past, I have had but few cases of mercurial disease consequent on my own treatment; and I am inclined to believe that, had a similar rule of practice been more generally adopted, the attempts lately made to cure syphilis without mercury, would have been superfluous." 187.

In the above sentiments we entirely agree, as they quadrate very completely with our own experience. We shall look forward for the continuation of Mr. Todd's remarks, and fail not to present an analysis of them to the readers of this Journal, as soon as they appear.

